# Complete Genome Sequence of Chinese Yam Necrotic Mosaic Virus from *Dioscorea opposita* in the Republic of Korea

**DOI:** 10.1128/genomeA.00778-16

**Published:** 2016-08-04

**Authors:** Joong-Hwan Lee, Chang-Gi Son, Joong-Bae Kwon, Hyo-Hun Nam, Yeongtae Kim, Su-Heon Lee, Fumei Zhao, Jae Sun Moon

**Affiliations:** a Institute for Bioresources Research, Gyeongsangbuk-do Agricultural Research and Extension, Andong, Republic of Korea; b School of Applied Biosciences, Kyungpook National University, Daegu, Republic of Korea; c Biosystems and Bioengineering Program, University of Science and Technology (UST), Daejeon, Republic of Korea

## Abstract

The complete genome sequence of Chinese yam necrotic mosaic virus (ChYNMV) consisting of 8,213 nucleotides containing one open reading frame was determined by the transcriptome data generated from *Discorea opposita*. This is the first report of the complete nucleotide sequence of ChYNMV from *Dioscorea opposita* in the Republic of Korea.

## GENOME ANNOUNCEMENT

In the Republic of Korea, yams (*Dioscorea opposita*) are cultivated mainly as a functional food and for medicinal purposes. More than 15 different viruses have been reported to infect *Dioscorea* species, including Chinese yam necrotic mosaic virus (ChYNMV) (1, 2). The host range of ChYNMV is restricted to *Dioscorea* spp. (3). The virus infection causes a range of symptoms, and results in economic losses due to lower yields and reduced quality of plant products. ChYNMV is a 660 nm long, flexuous, filamentous virus that is transmitted by aphids (4). ChYNMV was classified as a member of the genus *Macluravirus* (5) in the family *Potyviridae* by nucleotide sequencing of its genome. It has a single stranded RNA genome containing a long open reading frame encoding a polyprotein (6).

In August 2015, yam leaves showing virus disease symptoms were collected in Andong City, Gyeongsangbuk-do Province, Republic of Korea. Transcriptome data generated from the virus-infected leaves using a next-generation sequencing (NGS) method were analyzed. To construct a library with the TruSeq RNA sample prep kit (Illumina), total RNA was extracted from one pool of all *D. opposita* samples using TRI reagent (Molecular Research Center), and rRNA was removed using a Ribo-Zero rRNA removal kit (Epicentre). The size and purity of the constructed library were measured by BluePippin 2% agarose gel cassettes (Sage Science) and the Agilent 2100 Bioanalyzer (Agilent Technologies), respectively. Raw data were produced from the Illumina HiSeq 2500 paired-end RNA sequencing by the Theragen Etex Bio Institute (Suwon, South Korea). Raw data processing, including assembly of reads into contigs and annotation of contigs using the NCBI database for sequence similarity searches, was performed by SeqGenesis (Daejeon, South Korea). The resulting DNA sequences were assembled using Codoncode Aligner version 6.0 to produce a complete sequence. The assembled complete genome sequence consists of 8,213 nucleotides excluding the poly(A) tail and contains a polyprotein of 2,620 amino acids. The complete sequence (GenBank accession number KU641566, isolate CYNMV-BRI) was highly matched to Chinese yam necrotic mosaic virus isolate PES3 derived from Japan (GenBank accession number AB710145) with 8,224 nucleotides, showing 98% sequence identity and 100% query coverage. The isolate was constructed as an infectious clone of Chinese yam necrotic mosaic virus (3). On the other hand, the sequence alignment with ChYNMV isolate FX1 derived from China showed that the isolate CYNMV-BRI sequence was matched to the 3′ terminal portion of the whole-genome sequence.

In this work, we determined the complete sequence of the ChYNMV isolate CYNMV-BRI genome using transcriptome analysis of the *D. opposita* plant. To our knowledge, this is the first report of the complete nucleotide sequence of ChYNMV from *D. opposita* in the Republic of Korea.

### Accession number(s).

The complete genome sequence of Chinese yam necrotic mosaic virus isolate CYNMV-BRI has been deposited in GenBank under the accession number KU641566.

## References

[1] Zhang P, Peng J, Guo H, Chen J, Chen S, Wang J 2016 Complete genome sequence of yam chlorotic necrotic mosaic virus from *Dioscorea parviflora*. Arch Virol Jun 161:1715–1717. doi:10.1007/s00705-016-2818-7. 26973231

[2] Kenyon L, Shoyinka SA, Hughes Jd’A, Odu BO 2001 An overview of viruses infecting *Dioscorea yams* in sub-Saharan Africa, p 432–439. *In* Hughes JdA, Odu BO (ed), Plant virology in sub-Saharan Africa IITA, Ibadan, Nigeria.

[3] Kondo T, Fujita T 2012 Complete nucleotide sequence and construction of an infectious clone of Chinese yam necrotic mosaic virus suggest that macluraviruses have the smallest genome among members of the family *Potyviridae*. Arch Virol 157:2299–2307. doi:10.1007/s00705-012-1429-1. 22886278

[4] Kondo T, Kogawa K, Ito K 2015 Evaluation of cross protection by an attenuated strain of Chinese yam necrotic mosaic virus in *Chinese yam*. J Gen Plant Pathol 81:42–48. doi:10.1007/s10327-014-0561-z.

[5] Carstens EB, Ball LA 2009 Ratification vote on taxonomic proposals to the International Committee on Taxonomy of Viruses (2008). Arch Virol 154:1181–1188. doi:10.1007/s00705-009-0400-2. 19495937 PMC7086627

[6] Kondo T 2001 The 3′-terminal sequence of Chinese yam necrotic mosaic virus genomic RNA: a close relationship with macluravirus. Arch Virol 146:1527–1535. doi:10.1007/s007050170076. 11676415

